# Febrile infants without respiratory symptoms or sick contacts: are chest radiographs or RSV/influenza testing indicated?

**DOI:** 10.1186/s12879-021-06493-x

**Published:** 2021-08-23

**Authors:** Ali Ozcan, Evelyn Laskowski, Shashi Sahai, Kelly Levasseur

**Affiliations:** 1 Pediatric Emergency Department, Loma Linda Hospital, 11234 Anderson St, Loma Linda, CA 92354 USA; 2 William Beaumont Hospital, Royal Oak, MI USA; 3 Children’s Hospital of Michigan Detroit, Detroit, MI USA

**Keywords:** Febrile infants, Chest radiography, Respiratory syncytial virus, Influenza

## Abstract

**Background:**

Serious bacterial infection rates in febrile infants < 60 days are about 8–11%. Less than 1% of febrile infants with no respiratory symptoms will have pneumonia however, chest radiography (CXR) rates remain between 30 and 60%. Rapid Respiratory Syncytial Virus (RSV) and influenza (flu) testing is common, however, there is not enough data to determine if febrile infants without any respiratory symptoms should be tested. The goal of this study is to determine the rate of positive CXR and RSV/flu results in febrile infants with no respiratory symptoms and no sick contacts.

**Methods:**

Well-appearing febrile infants between 7 and 60 days of age who presented to the pediatric emergency department (PED) from September 1st, 2015 through October 30th, 2017 were enrolled. Demographic data, respiratory symptoms, CXR findings and RSV/flu results were collected. SAS statistical software was used for analysis.

**Results:**

129 infants met enrollment criteria. Of the 129 infants, 58 (45.0%) had no respiratory symptoms and no sick contacts. Of these 58, 36 (62.1%) received a CXR and none of them had any abnormal findings, 48 (82.8%) had RSV/flu testing, no patients tested positive for RSV and only one patient tested positive for flu. Costs of CXR and RSV/flu testing for this cohort was $19,788.

**Conclusion:**

The absence of positive CXRs in this patient population reinforces the current recommendations that CXR is not indicated. The low incidence of RSV/flu indicate that routine testing may not be necessary in this population especially outside of the flu season. Reduced testing could decrease overall costs to the healthcare system as well as radiation exposure to this population.

## Background

Febrile infants < 60 days of age are at high risk for serious bacterial infection (SBI) such as urinary tract infections, bacteremia or bacterial meningitis with rates of 8–11% [1, 2]. Multiple studies show that chest radiography (CXR) is not useful for evaluation of febrile infants with no respiratory symptoms, CXR rates are still between 30 and 60% [1, 3–8]. The Rochester criteria for evaluating febrile infants suggests that CXR may be indicated when there are findings of tachypnea, cough or focal abnormality on auscultation of the lungs [9]. Infants with no pulmonary symptoms are only at risk for pneumonia at a rate of 1% [4].

Although febrile infants < 60 days of age are at high risk for SBI, most illnesses are due to viral infection. Infants with viral infections are less likely to have an SBI. However, there is a 4–5% risk of a concomitant Respiratory Syncytial Virus (RSV) infection and urinary tract infection (UTI) [10]. Febrile infants with viral infections may not have viral symptoms including runny nose, cough, etc. [11]. There is limited data on when to perform viral testing (RSV/Influenza) on febrile infants without respiratory symptoms or sick contacts.

Our study is a sub-study of Project REVISE (Reducing Excessive Variability in Infant Sepsis Evaluation). As a sub-study of the project, we aimed to determine the number of positive CXR and RSV/flu in febrile infants with and without respiratory symptoms and sick contacts.

## Methods

### Protection of human subjects

The Institutional Review Boards (IRB) for Beaumont Health approved this study. Informed consent was waived (IRB number 2017-030). Project REVISE has received approval from the AAP Institutional Review Board.

### Setting

This was a retrospective cohort study and a sub-study of the Project REVISE data set at Beaumont Children’s Hospital, a tertiary hospital which has 24 pediatric emergency beds which annually sees around 23,500 patients, 50 inpatient pediatric beds, and 9 pediatric ICU beds. Project REVISE is a collaborative quality improvement project that aimed to improve and standardize care for febrile infants between the ages of 7–60 days. Pre-intervention data collected from September 2015 to August 2016 and post-intervention data was collected from December 2016 to November 2017. The intervention consisted of education to the residents, fellows and attending physicians in the ED and on the inpatient pediatric wards. One of the five metrics was to decrease the number of CXRs in infants without respiratory symptoms. Project REVISE involved 126 hospital teams across the country, specifically inpatient pediatric units and pediatric emergency departments. Physician investigators were tasked with educating their staff on evidence-based best practices in treatment and management of febrile infants in order to standardize care. Two of the main aims of the project included decreasing variation in care of febrile infants presenting to the ED and/or inpatient pediatric unit and decreasing unnecessary CXRs in febrile infants.

### Identification of febrile infants

All infants < 60 days of age with a Complete Blood Cell Count (CBC) who presented to the pediatric emergency department (PED) or were directly admitted to the pediatric floor from September 1st, 2015 through October 30th, 2017 were identified. Research Institute provided the listing of patients in the age range who had CBC's done. A retrospective chart review was performed by multiple reviewers (AO, EL, SS, KL) and infants were enrolled if they were between 7 and 60 days of age, well appearing and healthy as per the vitals and physical exam, and with documented or parent reported fever (Temp ≥ 38 °C or 100.4 °F).

Infants were excluded if they had any evidence of focal infection such as abscess, any chronic comorbid condition (e.g. congenital heart disease, neuromuscular disease, genetic/chromosomal abnormality, lung disease, etc.), ill-appearing, or admitted to an intensive care unit.

### Data collection

Demographic data (age, sex, gestational age) was collected as well as arrival temperature, upper respiratory infection symptoms (runny nose, cough, tachypnea), WBC (white blood cell count), platelet count, CXR findings, RSV/influenza PCR results and any documented sick contacts. All charts were reviewed manually by study personnel (AO, EL, SS, KL) including emergency history and physical exam and if an infant was admitted, all inpatient provider notes.

Cost data was derived from Beaumont Hospital Laboratory and Radiology Department. Raw costs of the tests were $255 for CXR and $221 for RSV/flu.

### Statistical analysis

Counts and frequencies were reported for categorical variables. Confidence intervals were calculated for the proportion of positive findings in the group of infants with both no symptoms and no documented sick contacts. Statistical analysis was performed using SAS statistical software Windows version 9.3.

## Results

### Study infants

**Fig. 1:**
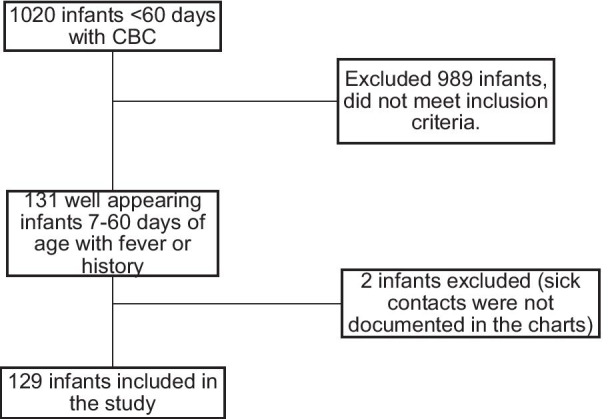
Patient inclusion and exclusion

1020 infants < 60 days of age had a CBC obtained and presented to our PED or pediatric floor during the study period. After chart review, 989 infants were excluded due to history findings not related with fever, they were ill appearing or had comorbidities. 129 infants were included in the study. 107 infants presented to the PED and 22 infants were admitted directly to the pediatric floor (Fig. 1). Mean age was 30.8 days, about half of the patients were male and most were white (Table 1). We see majority white patients at our institution. CXR and RSV/flu testing rates were not statistically significant between different races.

**Table 1:** Patient demographics

Characteristics	Percentage of patients n (%) n = 129
Sex	Male 65 (50.3%)
Race	White 85 (66%)
	African American 25 (19.3%)
	Other 19 (14.7%)
Primary language	English 126 (98%)
Gestational age	Full term (≥ 37 weeks) 124 (96.1%)
	Premature (33–37 weeks) 5 (3.9%)

### RSV/Flu and CXR results

Overall, 87.6% of the infants had RSV/Flu testing performed. All RSV/flu tests were done in the emergency department unless they were directly admitted then it was done upon arrival to the floor. 85/129 (65.9%) infants had no respiratory symptoms and 76/129 (58.9%) infants had no sick contacts. 58/129 (45.0%) infants had no respiratory symptoms and no sick contacts and all were documented as well appearing. 28 of the 53 infants with sick contacts had URI symptoms. URI symptoms divided as; only runny nose (11), only cough (8), runny nose and cough (20), tachypnea (2) and runny nose, cough, and tachypnea (3).

**Fig. 2:**
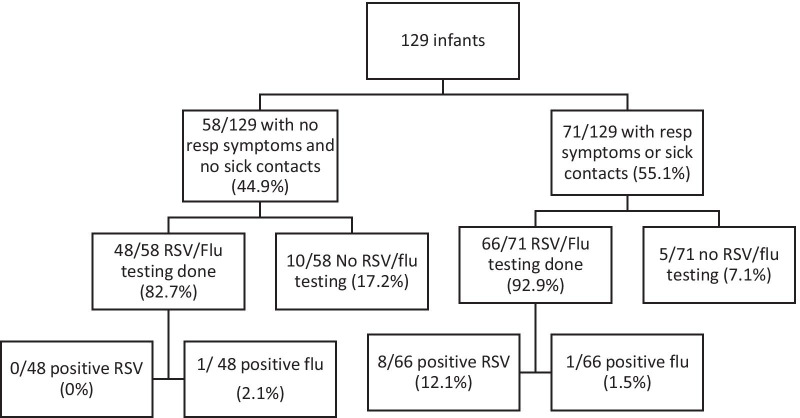
RSV/Flu testing

Of the 58 infants with no respiratory symptoms and no sick contact, 48 (82.8%) had RSV/flu testing and 26 (44.8%) had both CXR and RSV/flu testing. None tested positive for RSV (95% CI 0%, 7.4%) and 1 infant tested positive for flu (95% CI 0.4%, 10.9%) (Fig. 2).

71/129 (55.0%) infants had URI symptoms or sick contacts documented. Of the 71 infants, 66 (92.9%) were tested for RSV/flu with 1 positive for flu (1.5%) and 8 (12.1%) positive for RSV. 6 of the RSV positive infants had runny nose and cough, 1 had runny nose, cough and tachypnea and 1 only had runny nose. The infant with Flu had only cough. CXRs were performed on 49 of 71 with 2 of the 71 infants diagnosed with pneumonia.

**Fig. 3:**
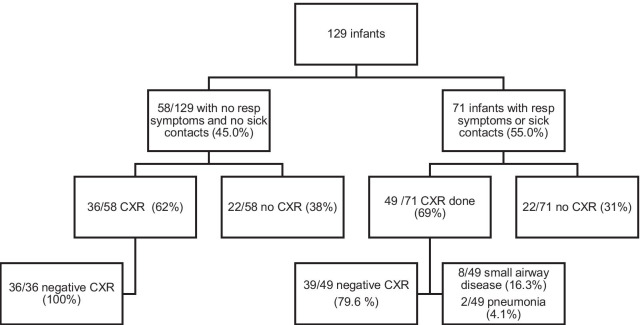
CXR results

Overall, CXR was performed on 65.8% of the infants. 36 of 58 (62.1%) with no respiratory symptoms and no sick contacts had a CXR all of which were negative for pneumonia. (95% CI 0%, 9.4%) (Fig. 3).

### Cost results

Costs were calculated with raw costs of the tests for each infant; $255 for CXR and $221 for RSV/flu. Eliminating testing for infants with no respiratory symptoms and sick contacts, the cost of the ED visits would decrease by $19,788. (CXR $255 × 36, RSV/flu $221 × 48).

## Discussion

The management of febrile infants 7–60 days old is highly variable [12]. Even though multiple studies show that CXR is not useful for evaluation of febrile infants with no respiratory symptoms, CXR rates are still between 30 and 60% this high rate might be explained by fear of missing an occult bacterial pneumonia [1, 4–8]. In total 65% of the infants had a CXR which is similar to previous studies [1, 4–6]. CXR rates did not differ in infants who had respiratory symptoms and sick contacts, and who had no respiratory symptoms and no sick contacts, 69% and 62%, respectively. All infants with no respiratory symptoms and no sick contact had a negative CXR which was similar to the results of the study by Bramson. They examined 361 febrile infants < 3 months of age with no pulmonary symptoms, none had a positive CXR [4]. Another study by Crain found that only 2 out of 148 infants with no respiratory symptoms had a positive CXR [7]. Sensitivity of having no respiratory symptoms to predict a negative CXR was 93% [7]. One study checked physician variations among pediatric emergency physicians and found that CXR rates were between 30 and 60% regardless of reported sick contacts which, in combination with respiratory symptoms, may be helpful to guide decision making [6].

Despite the low probability of a positive finding, many physicians order the CXR in fear of missing an occult pneumonia [13]. Rates of occult bacterial pneumonia in this population is about 1–3% [8]. One study showed the incidence of pneumonia in children < 12 months of age with high fever (> 39) without a source and WBC count greater than 20 × 10^9^/L was 7.8%. However, this study was limited to the selected children who had CXR and WBC ordered [14]. In 2003, ACEP (American College of Emergency Physicians) released an editorial mentioning that CXR should only be performed in infants who present with respiratory symptoms, abnormal pulse oximetry, hyperpyrexia, or marked leukocytosis [15]. Decreasing the rate of CXR in infants with no respiratory symptoms was one of the reasons our institution participated in Project REVISE.

There are no clear guidelines indicating when to perform RSV/flu testing in this population. Studies show that most febrile infants who were diagnosed with flu/RSV had either respiratory symptoms and/or sick contacts [10, 11, 16–18]. Identifying influenza and RSV may reduce antibiotic use and decrease unnecessary diagnostic testing [2].

Benito-Fernandez found that about 40% of all febrile infants < 3 months of age who were tested for flu during the flu season were positive. 75% had a sick contact and 46% had mild respiratory symptoms [11]. Another study showed that 34% of flu positive infants had no respiratory symptoms and the majority had history of a sick contact. Flu positivity rates were much higher during the peak season (up to 50%). One study also found decrease in antibiotic use in flu positive infants [16].

Smitherman evaluated the rate of SBI in flu positive infants 0–36 months of age and found that the odds of any SBI (excluding pneumonia) in the flu-negative group were 86% less than in those in the flu-positive group (OR (odds ratio): 0.14; 95% CI (Confidence Interval): 0.04–0.46) [17].

Febrile infants < 60 days of age with RSV infections were at significantly lower risk of SBI compared to the infants with negative RSV (rate of SBIs 7% vs 12.5%) but risk of UTI still remained significant [10]. The classic clinical presentation of bronchiolitis usually starts with URI and progresses to the lower respiratory tract over several days. Fever can be present in about a third of infants with bronchiolitis [18].

A systematic review was done to study the incidence of apnea in hospitalized infants with RSV bronchiolitis. When seriously ill patients were excluded, the incidence of apnea was between 1.2 and 4.3% [19]. Another study found that the frequency of apnea in infants < 12 months of age with bronchiolitis due to RSV and other viruses was 5.6% and one third of them presented with apnea as the first manifestation of bronchiolitis [20]. Infants with respiratory symptoms were at greater risk for apnea but the risk of apnea with no respiratory symptoms was not clear [19].

To our knowledge, there are no studies that look at the costs of CXR and RSV/flu testing in well-appearing febrile infants < 60 days of age. In a study done by Ziegler in 2010, they found the cost savings of not doing a CXR in adult trauma patients was approximately $103 resulting in an overall cost savings of $30,592 [21].

Amand calculated the healthcare resource use and cost of RSV infants across multiple age groups. They found that higher annual costs in the RSV infants compared to the matched controls across all age groups; ranging from $7,535 to $40,405 [22]. None of the well-appearing infants < 60 days of age with no sick contacts and respiratory symptoms tested positive for RSV. Although, RSV causes significant cost to healthcare, cost may be decreased by not testing this population for RSV/flu.

One of the inherent limitations of this study was the retrospective nature of the data since we relied on history and physical examination findings documented in the chart. Identification of the cohort was limited to infants for whom a CBC was performed in the PED or pediatric floor. Although it is standard of care to obtain a CBC in febrile infants we may have missed patients if they did not have the standard work-up. One of the other limitations was that our rate of CXR was 62% at the start of this REVISE study and we were actively trying to reduce the number of CXRs during the study period, which might have affected the rate of CXRs. Also, the sample size was not large enough to ensure power and the wide 95% CI around the estimates due to small sample size to conclude there is no necessity of CXR and RSV/flu testing in febrile infants with no respiratory symptoms and no sick contacts.

## Conclusion

This study reconfirms the current guidelines that state a CXR is not necessary in this population. RSV/flu testing may not need to be obtained in the work up of well appearing febrile infants with no URI symptoms and no sick contacts especially outside of the flu season. Larger studies need to be done to determine the cost benefit to this population.

## Data Availability

Data sheet is available upon request and submitted with the manuscript.

## Abbreviations

CXR: Chest radiography
RSV: Respiratory Syncytial Virus
Flu: Influenza
PED: Pediatric emergency department
SBI: Serious bacterial infection
UTI: Urinary tract infection
REVISE: Reducing Excessive Variability in Infant Sepsis Evaluation
CBC: Complete blood cell count
WBC: White blood cell count
ACEP: American College of Emergency Physicians
OR: Odds ratio
CI: Confidence interval
IRB: Institutional Review Boards
AO: Ali Ozcan
EL: Evelyn Laskowski
SS: Shashi Sahai
KL: Kelly Levasseur
